# Megalin Knockout Reduces SGLT2 Expression and Sensitizes to Western Diet-induced Kidney Injury

**DOI:** 10.1093/function/zqae026

**Published:** 2024-05-20

**Authors:** Elynna B Youm, Katherine E Shipman, Wafaa N Albalawy, Amber M Vandevender, Ian J Sipula, Youssef Rbaibi, Allison E Marciszyn, Jared A Lashway, Emma E Brown, Corry B Bondi, Cary R Boyd-Shiwarski, Roderick J Tan, Michael J Jurczak, Ora A Weisz

**Affiliations:** Renal-Electrolyte Division, University of Pittsburgh School of Medicine, Pittsburgh, PA 15261, USA; Department of Human Genetics, University of Pittsburgh School of Public Health, Pittsburgh, PA 15261, USA; Renal-Electrolyte Division, University of Pittsburgh School of Medicine, Pittsburgh, PA 15261, USA; Renal-Electrolyte Division, University of Pittsburgh School of Medicine, Pittsburgh, PA 15261, USA; Department of Human Genetics, University of Pittsburgh School of Public Health, Pittsburgh, PA 15261, USA; Division of Endocrinology and Metabolism, Department of Medicine, University of Pittsburgh School of Medicine, Pittsburgh, PA 15261, USA; Division of Endocrinology and Metabolism, Department of Medicine, University of Pittsburgh School of Medicine, Pittsburgh, PA 15261, USA; Renal-Electrolyte Division, University of Pittsburgh School of Medicine, Pittsburgh, PA 15261, USA; Renal-Electrolyte Division, University of Pittsburgh School of Medicine, Pittsburgh, PA 15261, USA; Division of Endocrinology and Metabolism, Department of Medicine, University of Pittsburgh School of Medicine, Pittsburgh, PA 15261, USA; Renal-Electrolyte Division, University of Pittsburgh School of Medicine, Pittsburgh, PA 15261, USA; Renal-Electrolyte Division, University of Pittsburgh School of Medicine, Pittsburgh, PA 15261, USA; Renal-Electrolyte Division, University of Pittsburgh School of Medicine, Pittsburgh, PA 15261, USA; Renal-Electrolyte Division, University of Pittsburgh School of Medicine, Pittsburgh, PA 15261, USA; Division of Endocrinology and Metabolism, Department of Medicine, University of Pittsburgh School of Medicine, Pittsburgh, PA 15261, USA; Renal-Electrolyte Division, University of Pittsburgh School of Medicine, Pittsburgh, PA 15261, USA

**Keywords:** metabolism, proximal tubule, glucose tolerance

## Abstract

Megalin (*Lrp2*) is a multiligand receptor that drives endocytic flux in the kidney proximal tubule (PT) and is necessary for the recovery of albumin and other filtered proteins that escape the glomerular filtration barrier. Studies in our lab have shown that knockout (KO) of *Lrp2* in opossum PT cells leads to a dramatic reduction in sodium–glucose co-transporter 2 (SGLT2) transcript and protein levels, as well as differential expression of genes involved in mitochondrial and metabolic function. SGLT2 transcript levels are reduced more modestly in *Lrp2* KO mice. Here, we investigated the effects of *Lrp2* KO on kidney function and health in mice fed regular chow (RC) or a Western-style diet (WD) high in fat and refined sugar. Despite a modest reduction in SGLT2 expression, *Lrp2* KO mice on either diet showed increased glucose tolerance compared to control mice. Moreover, *Lrp2* KO mice were protected against WD-induced fat gain. Surprisingly, renal function in male *Lrp2* KO mice on WD was compromised, and the mice exhibited significant kidney injury compared with control mice on WD. Female *Lrp2* KO mice were less susceptible to WD-induced kidney injury than male *Lrp2* KO. Together, our findings reveal both positive and negative contributions of megalin expression to metabolic health, and highlight a megalin-mediated sex-dependent response to injury following WD.

## Graphical Abstract

**Graphical Abstract:**
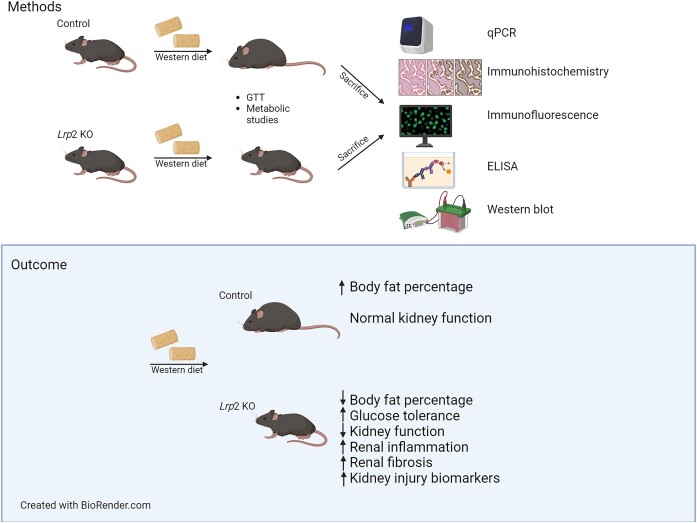

## Introduction

The Western-style diet (WD) is characterized by highly processed food rich in sugar, salt, animal protein, and saturated and *trans*-fats. The average annual high-fructose corn syrup intake among Americans has increased over 120-fold over the past several decades and the prevalence of obesity has nearly doubled.^1^,^2^ Obesity and diets high in processed meat and refined sugars promote the development of metabolic syndrome and chronic diseases such as cardiovascular disease and type 2 diabetes (T2D).^3^,^4^

The kidney has an extraordinary metabolic rate estimated to be double that of the liver and brain.^5^ The majority of this energy expenditure supports transport by the proximal tubule (PT) to maintain salt and water homeostasis. Contrary to other nephron segments, almost no glycolysis occurs in the PT; this pathway accounts for only 4% of ATP produced under aerobic conditions.^6^ Rather, PT cells utilize primarily lactate, pyruvate, glutamate, and free fatty acids as energy sources to drive oxidative phosphorylation.^7^ In addition to maintaining its own energy needs, gluconeogenesis driven by the PT contributes roughly a quarter of serum glucose in rats under normal conditions. Under starvation conditions, this increases to nearly half of total blood glucose. It has also been shown that the PT is equally important in maintaining systemic gluconeogenesis in humans.^8^,^9^

In addition to generating glucose, the PT is also responsible for reabsorbing glucose from the filtered plasma to prevent its excretion in the urine. Glucose is reabsorbed via luminal sodium–glucose co-transporters 2 (SGLT2) and 1 (SGLT1), which are differentially expressed in early and later segments of the PT, respectively. SGLT2 is a low-affinity high-capacity transporter that normally reabsorbs ∼90% of filtered glucose, while the high-affinity low-capacity SGLT1 reabsorbs the remaining ∼10%. Rather than serving as metabolic fuel for PT cells, glucose is exported to the bloodstream via basolateral Glucose transporter 2 (GLUT2) receptors.

Inhibition of SGLT2 by phlorizin-related derivatives has become a powerful approach to maintain glycemic control in patients with T2D. These drugs prevent the capture of filtered glucose by the PT, resulting in reduced plasma glucose levels. Strikingly, numerous clinical trials have concluded that SGLT2 inhibitors (SGLT2is) have benefits beyond glucose regulation, including improved outcomes in heart failure patients and slower progression of chronic kidney disease in patients with or without T2D.^10^^-^ ^14^ The mechanisms behind these protective effects are not well understood but have been suggested to include reduced mitochondrial burden in PT cells resulting from lower transport needs and reduced intraglomerular pressures and reduction in proteinuria.^15^,^16^

In addition to glucose, the PT also recovers essentially all proteins that escape the glomerular filtration barrier to prevent their excretion in the urine. Proteins are internalized upon binding to the multiligand receptors megalin (*Lrp2*) and cubilin and efficiently targeted to lysosomes for degradation.^17^,^18^ Megalin is a >600 kDa member of the low-density lipoprotein (LDL) receptor family that contains 4 ligand-binding regions, a single transmembrane domain, and a cytoplasmic tail with binding motifs that engage the clathrin adaptor protein *Dab2*.^17^Beyond its function as a receptor, *Lrp2* plays a unique role in driving membrane flux through the endocytic pathway.^19^,^20^ While the expression and subcellular distribution of endocytic markers appear unchanged in *Lrp2* knockout (*Lrp2* KO) cells, trafficking through endocytic compartments is dramatically reduced when megalin is absent or its function is impaired.^19^^-^ ^21^

Megalin expression clearly impacts kidney function, but its complex effects on the development and progression of kidney disease have been challenging to disentangle. In contrast, selective kidney deletion of *Lrp2* in mice results in a more modest phenotype, characterized primarily by urinary excretion of low molecular weight proteins.^22^^-^ ^24^ Impairing megalin expression or function reduces the uptake of nephrotoxic drugs and limits their oxidative damage.^25^,^26^ On the other hand, preserving megalin expression by inhibiting proprotein convertase subtilisin/kexin type 9 in proteinuric animals is protective in dampening renal fibrosis and other injury markers.^27^ The expression of megalin is affected in diseases such as T2D, with unknown consequences to disease progression.^28^,^29^

Recently, our laboratory made the surprising discovery that KO of *Lrp2* in a highly differentiated PT cell line causes a dramatic (>80%) reduction in SGLT2 (*Slc5a2*) mRNA transcripts. A more modest effect was observed in a kidney selective *Lrp2* KO mouse model.^21^ The transcriptional regulation of SGLT2 by megalin suggested the possibility that *Lrp2* KO may recapitulate the glucose tolerance and/or cardiac and renal protective effects of SGLT2is.

The WD has been associated with an increased incidence of T2D, tubular injury, and chronic kidney disease in humans.^30^,^31^ We hypothesized that reduced expression of SGLT2 in *Lrp2* KO mice would confer higher glucose tolerance, similar to that seen in SGLT2 (*Slc5a2*) KO animals and in wild-type mice treated with SGLT2is.^32^,^33^

## Materials and Methods

### Animal Care and Use

Mouse breeding and long-term monitoring experiments were approved by the Institutional Animal Care and Use Committee (IACUC), University of Pittsburgh (#19095734). *Lrp2 lox/lox* mice,^22^ originally obtained from Thomas Willnow (Max Delbrück Center for Molecular Medicine), were bred to mice expressing *Cre* recombinase driven by the central nervous system- and kidney-specific *EMX* promoter [generously provided by Cecilia Lo (University of Pittsburgh)]. *Lrp2* KO and control mice were established by heterozygous breeding. Control mice were defined as mice with genotype *Lrp2*^lox/lox^  *Cre*^−/−^, and experimental *Lrp2* KO mice were defined as mice with genotype *Lrp2*^lox/lox^  *Cre*^−/+^. Male mice were fed a regular chow (RC) diet (ProLab IsoPro RMH 3000; kcal provided as approximately 26% protein, 14% fat, and 60% carbohydrate, with 0.26% sodium). Following initial glucose tolerance tests (GTTs) and metabolic studies, mice were placed on a WD [purchased from Research Diets (RD Western Diet, D12079B) and provided kcal as approximately 17% protein, 40% fat (44% saturated and 56% unsaturated), and 43% carbohydrate (70% sucrose, 30% starch), with 0.26% sodium] for 9 wk prior to GTTs and metabolic studies. Body weight and changes in health condition were monitored weekly. Mice were maintained on WD for an additional 3 wk prior to sacrifice and collection of urine, tissue, and blood samples. The initial study was performed on a cohort of 19 male mice (11 control and 8 *Lrp2* KO mice; 8-11 wk at the start of study). To investigate sex-specific differences, the effect of WD was evaluated in a separate study on a cohort of 8 female mice (5 control and 3 *Lrp2* KO mice; 23 wk old at the start of study, placed on WD for 4 wk prior to GTT and metabolic studies, and maintained on WD for an additional 4 wk prior to sacrifice). Prior to sacrifice, 4 male control, 2 male *Lrp2* KO, and 1 female control mouse died.

### Metabolic Studies

Major determinants of whole-body energy balance were assessed in the Sable Systems Promethion Multiplexed Metabolic Cage System. Mice were individually housed in a home cage setting for 72 h, during which feeding, activity, energy expenditure, drinking, and respiratory exchange ratio (RER) were continuously monitored. The first 24 h were considered acclimation and not included in the analysis, such that data shown represent 48 h of data beginning on day 2 of housing. Body composition was measured by EchoMRI. GTTs were performed after a 6-h morning fast (7 am-1 pm). Following a collection of basal blood sample (*t* = 0) by tail bleed, mice received an intraperitoneal bolus injection of glucose at 2.0 mg/kg body mass on RC, and 1.5 mg/kg body mass on WD.

### Western Blotting

Kidney samples were immediately frozen in liquid nitrogen after collection and stored at −80°C. Kidneys were lysed in radioimmunoprecipitation assay (RIPA) buffer or Cell Lytic MT Cell Lysis Reagent (Sigma C3228), with protease and phosphatase inhibitors (5 µg/mL leupeptin, 7 µg/mL pepstatin A, 1 m m phenylmethylsulfonyl fluoride, Complete Protease Inhibitor EDTA-Free (Roche, 04693159001; 1 tablet/10 mL of buffer), and PhosSTOP (Roche, 04906837001; 1 tablet/10 mL of buffer). Protein concentration was measured using a BCA Protein Assay Kit (Pierce 23227) and equivalent amounts of total protein were separated by sodium dodecyl sulfate–polyacrylamide gel electrophoresis (SDS-PAGE). All blots were imaged using the ChemiDoc Touch Imaging System (Bio-Rad, Hercules, CA, USA). Band intensities were quantified using Bio-Rad Image Lab software. Megalin was detected with anti-megalin antibody from Santa Cruz (sc-515750, 1:1000) and SGLT2 was detected with anti-SGLT2 antibody from Proteintech (24654-1-AP, 1:500).

### Indirect Immunofluorescence

Kidneys were fixed in formalin overnight at 4°C and then transferred to 70% ethanol. Samples were sent to the University of Pittsburgh Biospecimen Core (PBC) for paraffin embedding and sectioning. Sections were deparaffinized using a standard protocol with xylene, 100% ethanol, and 95% ethanol. A double/sequential labeling protocol was used to stain deparaffinized kidney sections with primary antibodies from the same host species.^34^ Briefly, sections were rehydrated using Tris-buffered saline with 0.05% Tween-20 (TBST) and antigen retrieval was performed using heat and antigen unmasking solution (Vector, H-3300-25). Subsequently, samples were blocked in TBST containing 10% goat serum (TBST-Ser) for 1 h, and then incubated in TBST-Ser with rabbit anti-SGLT2 (1:250, ab85626) overnight. On the following day, samples were washed 3 times for 5 min each with TBST and incubated in anti-rabbit Alexa Fluor 488 in TBST-Ser for 30 min, washed 3 more times for 5 min each in TBST, and blocked in goat anti-rabbit unconjugated F(ab) fragments in TBST-Ser for 15 min. Samples were then incubated in TBST-Ser with rabbit anti-megalin antibody generously provided by Dr Daniel Biemsderfer and Dr Peter Aronson (Yale University, 1:1000, MC-220) for 2 h, washed 3 times for 5 min each in TBST, and incubated in anti-rabbit Alexa Fluor 647 in TBST-Ser for 30 min. After washing 3 times for 5 min each in TBST, coverslips were mounted onto the slides with Prolong Glass + NucBlue (Invitrogen, P36981) and sealed using clear nail polish. Samples were imaged using a Leica STELLARIS 8 inverted confocal microscope using a 63× oil immersion objective.

### Blood Chemistry Analysis

Blood was collected at the time of sacrifice, and samples were inserted in iSTAT CHEM8+ cartridges (Abbot) and analyzed using a handheld iSTAT 1 blood analyzer (Abbot).

### Histology and Injury Scoring

Formalin-fixed kidneys were transferred to 70% ethanol and sent to the University of Pittsburgh Biospecimen Core for paraffin embedding, sectioning, and Hematoxylin and eosin (H&E) and Masson’s Trichrome staining. The entirety of the cortex and corticomedullary regions of a single transverse stained section at the midpoint of the kidney was imaged using a Leica LAS X Widefield microscope and scored by an investigator blinded to sample group according to the following scale: 0 = no injury, 1 = 1%-25% of tissue has injury, 2 = 26%-50% of tissue has injury, 3 = 51%-75% of tissue has injury, and 4 = 76%-100% of tissue has injury. Injury was defined as any tubular injury (dilation, sloughing, vacuolization) or inflammation.

### Quantitative Real-Time Reverse Transcriptase Polymerase Chain Reaction

Kidney samples were immediately frozen in liquid nitrogen and stored at −80°C. Kidneys were lysed using TRIzol Reagent (Ambion, Carlsbad, CA, USA) to isolate RNA. cDNA was generated with the RevertAid Reverse Transcriptase Kit (ThermoFisher Scientific, Pittsburgh, PA, USA). The quantitative real-time reverse transcriptase polymerase chain reaction (qRT-PCR) used iTAQ Universal SYBR Green Supermix (Bio-Rad Laboratories). Primer sets are listed in Table S1. Results were normalized to *Ppia* (cyclophilin A), and expression was determined using the comparative 2(−ΔΔC(T)) method.^35^,^36^

## Results

### Expression of SGLT2 in Control and *Lrp2* KO Mice

**Figure 1.**
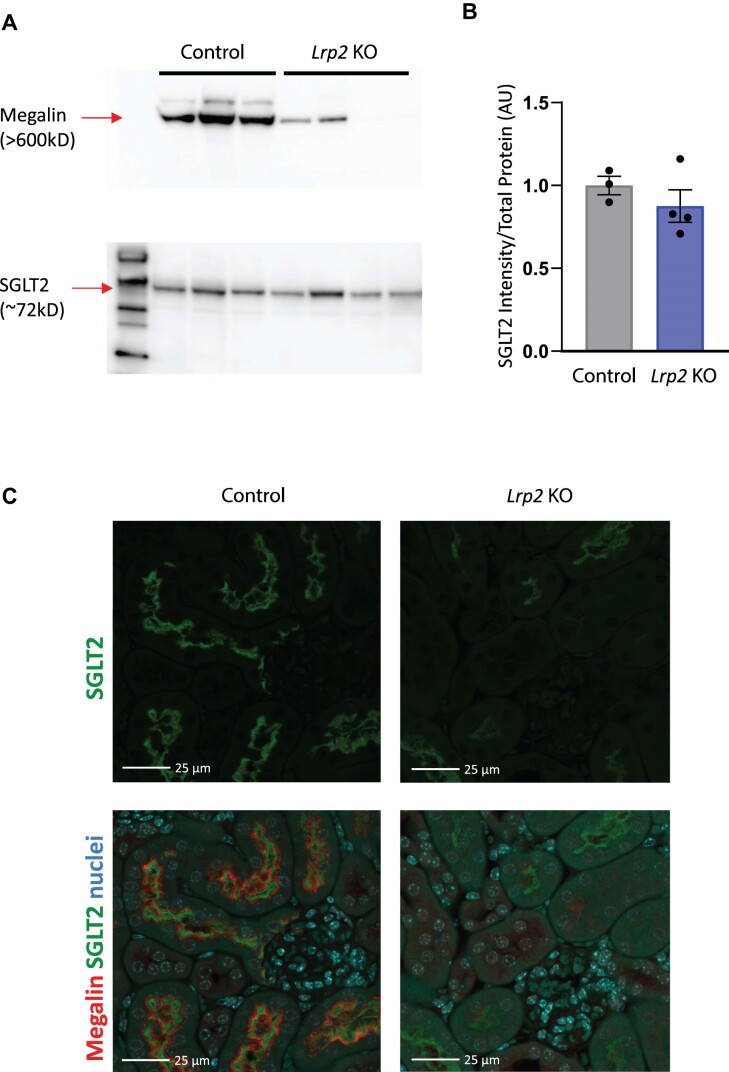
*Lrp2* KO mice have reduced levels of SGLT2. Western blot (A) of mouse kidney lysates from control and *Lrp2* KO male mice on regular chow probed to detect megalin and SGLT2. (B) Quantitation of SGLT2 expression in panel A. Data analyzed by an unpaired *t*-test. (C) Cortical kidney sections of control and *Lrp2* KO mice were stained to reveal SGLT2 (green). Merged panels below each image show megalin (red) and nuclei (blue) to verify efficient KO. SGLT2 exposures and processing were identical for all images. Megalin staining intensity in the *Lrp2* KO image was selectively enhanced to enable better comparison of expressing versus non-expressing cells. Scale bar: 25 µm.

Western blotting and indirect immunofluorescence were used to confirm highly efficient (>90%) KO of megalin in EMX-Cre *Lrp2* KO male mice compared with control mice (Figure 1A, top panel). By western blotting, we observed only a modest decrease (∼13%) in SGLT2 protein expression compared to their control counterparts (Figure 1A [bottom panel] and B). Indirect immunofluorescence staining of kidney sections revealed a more pronounced reduction in SGLT2 expression in *Lrp2* KO mice compared with controls (Figure 1C).

### Glucose Tolerance and Metabolic Function in Control and *Lrp2* KO Mice

**Figure 2.**
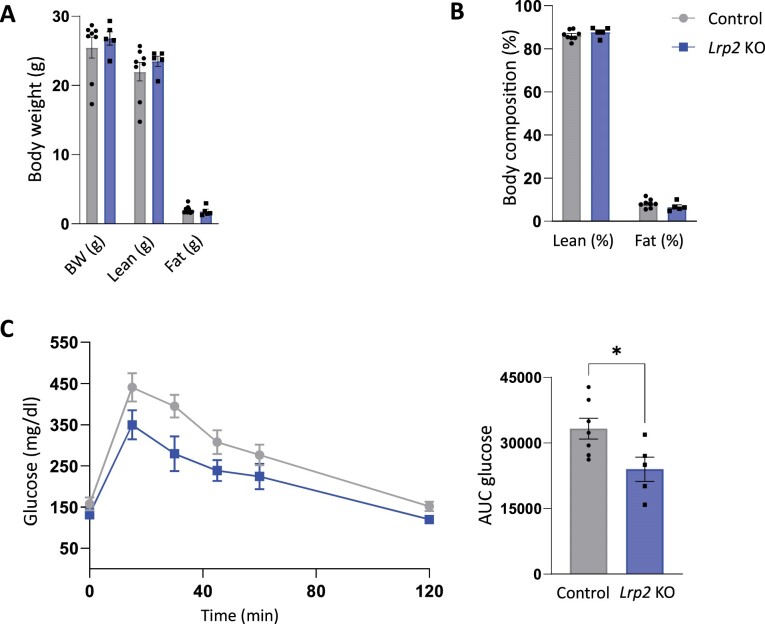
*Lrp2* KO mice have improved glucose homeostasis on regular chow. Body weight (A) and composition (B) control and *Lrp2* KO mice are plotted. Data analyzed by an unpaired *t*-test with multiple comparisons correction (Holm-Sidak). (C) Temporal changes in plasma glucose following intraperitoneal injection of 2.0 mg/kg glucose in fasted control and *Lrp2* KO mice. Area under the curve (AUC) for glucose calculated in panel C is shown in panel D. (*P*-values denoted by asterisk: *≤0.05.) Data analyzed by an unpaired *t*-test.

*Lrp2* KO male mice fed RC had baseline body weights, total fat mass, and total lean mass similar to their control counterparts (Figure 2A and B). We tested the effect of *Lrp2* KO on glucose tolerance in mice on RC diets. Despite the modest reduction in SGLT2 expression, *Lrp2* KO mice cleared a bolus of injected glucose more rapidly than their control counterparts (Figure 2C and D). The magnitude of this effect is comparable to that previously reported in SGLT2 KO mice.^32^

**Figure 3.**
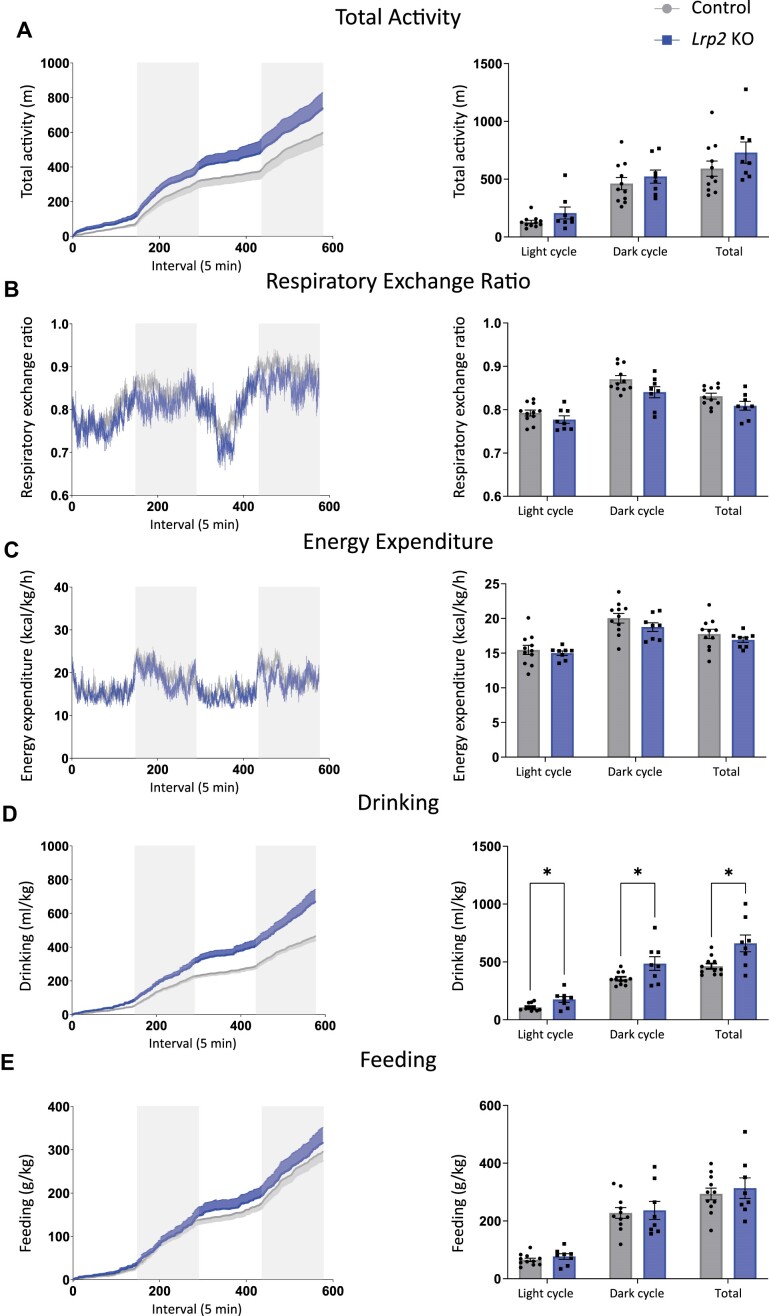
Metabolic parameters of control and *Lrp2* KO mice on regular chow. Control and *Lrp2* KO mice were evaluated in metabolic cages for 48 h. Traces for each parameter are shown in the left graph of each panel (gray bars denote dark cycles), and average data for each cycle and total over the 48 h period are plotted on the right. (A) Activity; (B) respiratory exchange ratio; (C) energy expenditure; (D) drinking; and (E) feeding. (*P*-values denoted by asterisk: *≤0.05.) Data analyzed by an unpaired *t*-test with multiple comparisons correction (Holm-Sidak).

Following GTTs, mice were placed in metabolic cages and metabolic parameters (total activity, RER, energy expenditure, drinking, and feeding) were measured over a 48 h period. There were no significant differences in metabolic parameters between *Lrp2* KO mice and control mice (Figure 3A-C and E), except that *Lrp2* KO mice drank significantly more than control mice (Figure 3D). This increase in water intake is consistent with observations in SGLT2 KO mice and in wild-type mice treated with SGLT2i.^32^,^37^ The average RER was also lower in *Lrp2* KO mice compared to controls but did not reach significance, with *P*-values of 0.181, 0.177, and 0.177 for the light, dark, and total cycles, respectively (Figure 3B).

### Glucose Tolerance and Metabolic Function in Control and *Lrp2* KO Mice Following a WD

**Figure 4.**
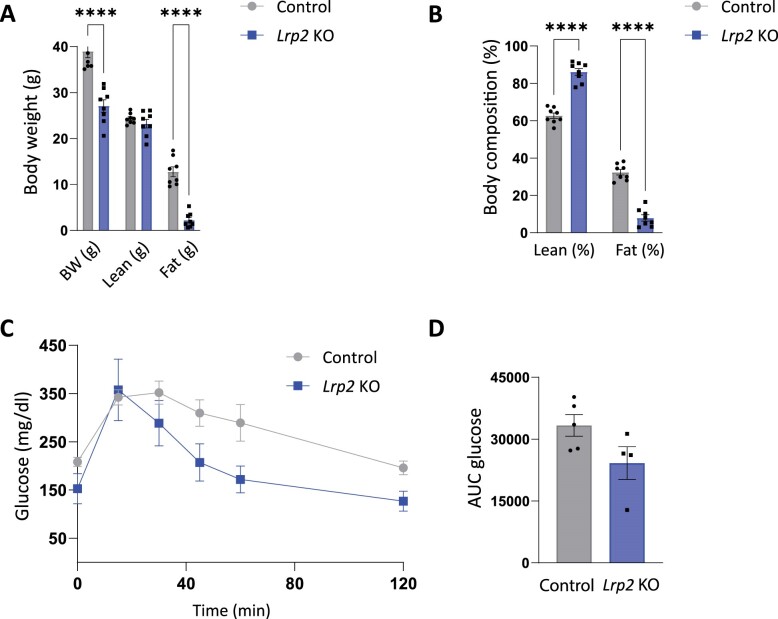
Body composition and glucose homeostasis are altered in *Lrp2* KO mice after Western diet. Control and *Lrp2* KO mice were placed on a Western diet for 9 wk and analyzed as in Figure 2. Body weight (A) and composition (B) of control and *Lrp2* KO mice are plotted. Data analyzed by an unpaired *t*-test with multiple comparisons correction (Holm-Sidak). (C) Temporal changes in plasma glucose following intraperitoneal injection of 1.5 mg/kg glucose in fasted control and *Lrp2* KO mice. AUC for glucose calculated in panel C is shown in (D). (*P*-values denoted by asterisk: ^****^≤0.0001.) Data analyzed by an unpaired *t*-test.

**Figure 5.**
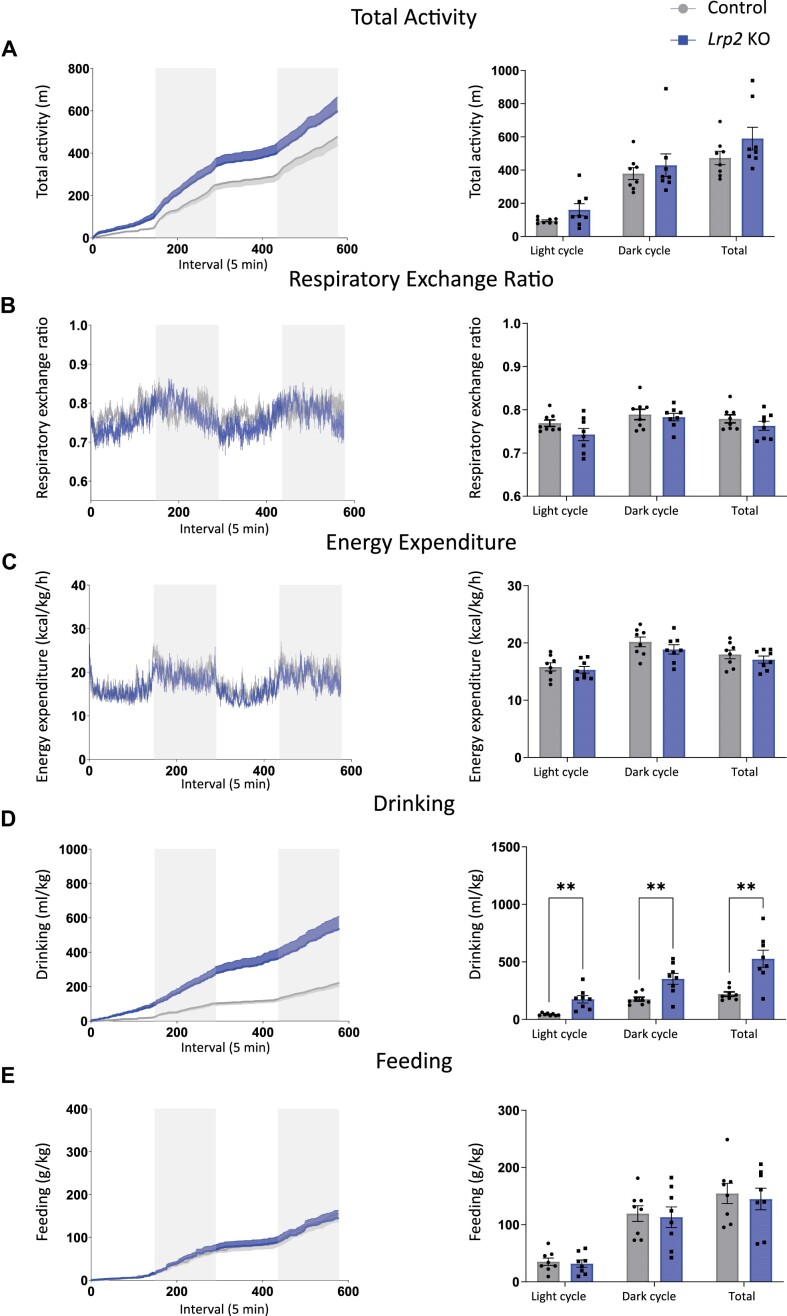
Metabolic parameters of control and *Lrp2* KO mice after Western diet. Control and *Lrp2* KO mice fed a Western diet for 9 wk were evaluated in metabolic cages for 48 h. Traces for each parameter are shown in the left graph of each panel (gray bars denote dark cycles), and average data for each cycle and total over the 48 h period are plotted on the right. (A) Activity; (b) respiratory exchange ratio; (c) energy expenditure; (d) drinking; and (e) feeding. (*P*-values denoted by asterisk: **≤0.01.) Data analyzed by an unpaired *t*-test with multiple comparisons correction (Holm-Sidak).

Following their initial evaluation on RC, mice were fed a WD high in fat and refined sugar with normal NaCl concentration for 9 wk and evaluated as before. Remarkably, unlike control mice, which gained roughly 50% of their original body weight, *Lrp2* KO mice did not gain weight on WD. Moreover, *Lrp2* KO mice on WD had significantly lower % of fat compared to control counterparts (Figure 4A and B). Similar to our observations in mice on RC, glucose clearance in *Lrp2* KO mice fed a WD was more rapid than in control mice fed a WD, with average area under the curve approaching significance (*P* = 0.086) (Figure 4C and D). Also consistent with their behavior on RC, *Lrp2* KO mice on WD exhibited no significant differences in metabolic parameters compared to control mice (Figure 5A-C and E), except for a significant increase in water intake (Figure 5D).

### Kidney Injury in *Lrp2* KO Mice Fed a WD

**Figure 6.**
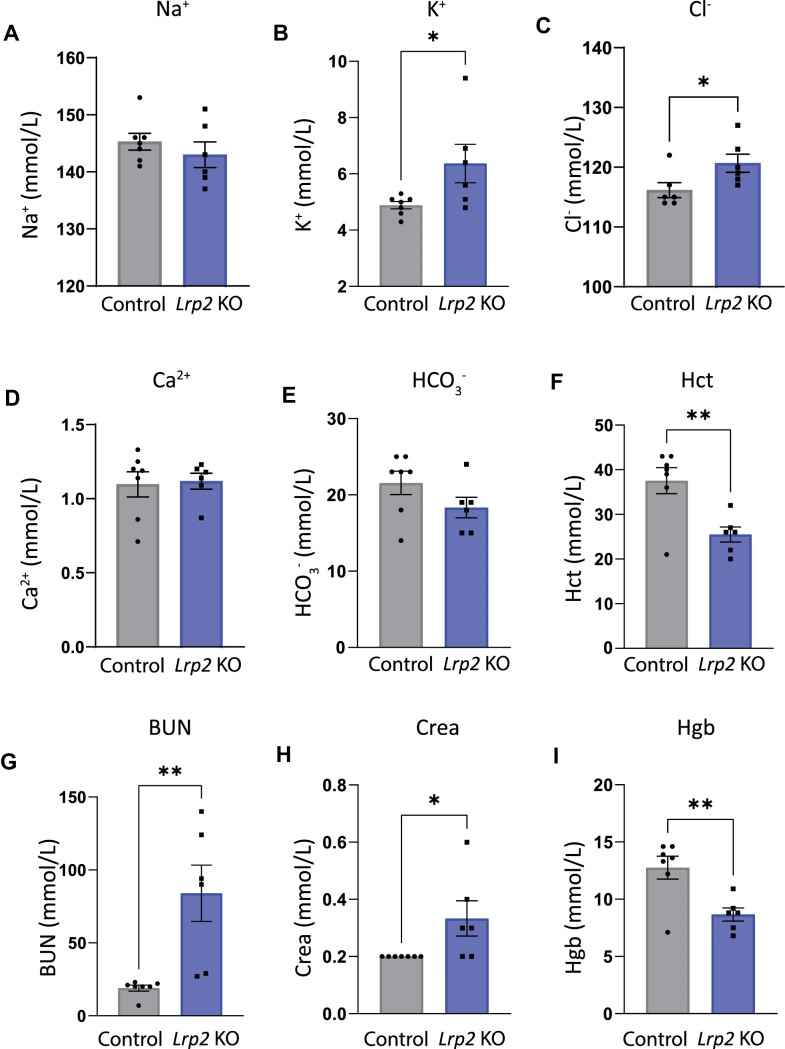
Blood chemistry analysis of *Lrp2* KO and control mice after Western diet. Blood was collected from control and *Lrp2* KO mice sacrificed after Western diet and analyzed by iSTAT to measure (A) Na^+^; (B) K^+^; (C) Cl^−^; (D) NaHCO_3_^−^; (E) Ca^2+^; (F) hemoglobin (Hb); (G) blood urea nitrogen (BUN); (H) creatinine; and (I) hematocrit (Hct) levels. (*P*-values denoted by asterisks: *≤0.05, **≤0.01.) Data analyzed by an unpaired *t*-test, except for creatinine, which was analyzed by a rank sum test.

Male mice fed WD were sacrificed, and their blood chemistry was compared with age-matched *Lrp2* KO and control mice fed RC (Figure 6 and Table 1). Blood chemistry of *Lrp2* KO mice fed RC was indistinguishable from age-matched control mice (Table 1). Similarly, iSTAT values for control mice on WD (Figure 6) were unchanged compared with mice fed RC (Table 1). By contrast, *Lrp2* KO mice on WD had significantly increased levels of K^+^ and Cl^−^, as well as higher blood urea nitrogen (BUN), and creatinine levels, all suggestive of kidney injury (Figure 6). Hematocrit and hemoglobin levels were reduced in *Lrp2* KO mice compared to control mice. The lack of hemoconcentration suggests that volume depletion was unlikely to be driving their aberrant blood ion levels (Figure 6).

**Table 1.** iSTAT Blood Chemical Analysis of Control and *Lrp2* KO Mice on Regular Chow

Average iSTAT values	*n*	Na^+^ (mmol/L)	K^+^ (mmol/L)	Cl^−^ (mmol/L)	HCO_3_ (mmol/L)	Ca^2+^ (mmol/L)	Hgb (g/dL)	BUN (mmol/L)	Crea (mg/dL)	Hct (%)
Male control	3	146.7 [1.475]	4.133 [0.1299]	116.7 [1.249]	22 [1.541]	1.12 [0.8518]	11.9	29.67 [2.047]	<0.2	35 [2.910]
Male KO	4	146.8 [2.236]	4.4 [0.6864]	113.5 [1.520]	24 [1.358]	1.193 [0.0535]	12.15	25.75 [19.28]	<0.2	35.75 [1.708]

Values are means. No significant differences detected by an unpaired *t*-test. SEM noted in brackets.

**Figure 7.**
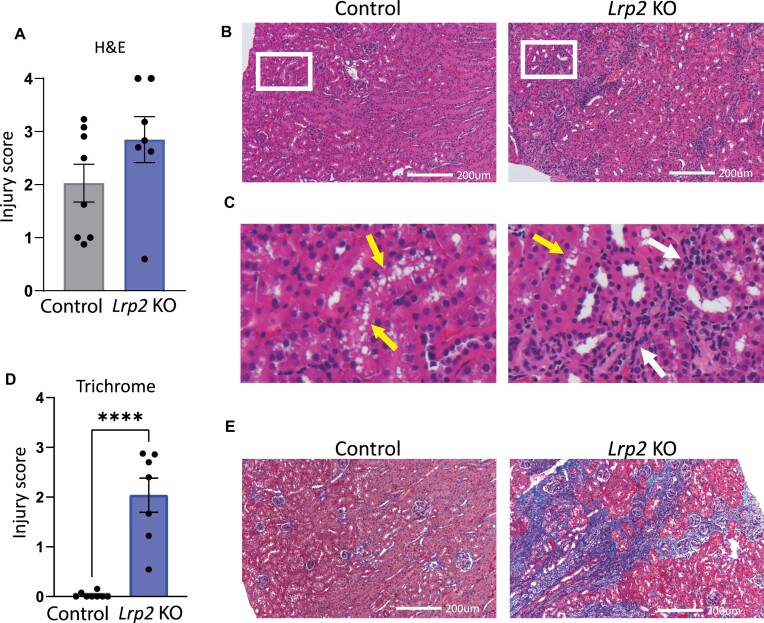
*Lrp2* KO mice have exacerbated kidney injury after Western diet. H&E and Masson’s Trichrome stained sections were scored in a blinded manner as described in the “Methods” section. (A) H&E scoring of stained sections of mice on WD. (B) Representative H&E stained kidney sections from control and *Lrp2* KO mice after Western diet. Zoomed-in images of areas denoted with red box in (B) are enlarged in (C). Areas of proximal tubule vacuolization (yellow arrows) and inflammatory responses (black arrows) are shown. (D) Masson’s Trichrome scoring of stained kidney sections from mice on WD. (*P*-values denoted by asterisks: ^****^≤0.0001.) Data analyzed by an unpaired *t*-test. (E) Representative Masson’s Trichrome (E) stained kidney sections from control and *Lrp2* KO mice after Western diet. All images shown are from mice with injury scores near the mean of their group.

To further evaluate renal injury, kidney sections from sacrificed male mice were processed for histologic staining and injury level scored. Examination of H&E-stained kidney sections from mice on RC revealed slightly greater baseline injury in *Lrp2* KO mice compared with controls (Figure S1). After WD, control male mice developed enlarged tubular vacuoles, consistent with previous observations in wild-type or control mice fed a high-fat diet^38^,^39^ (Figure 7A-C). By contrast, male *Lrp2* KO mice had fewer tubular vacuoles, but exhibited dramatically increased inflammation (Figure 7A-C). Reduced vacuolization in response to WD has also been reported in Sglt2i-treated mice.^40^ Changes in renal fibrosis were more striking, with *Lrp2* KO mice on WD exhibiting significantly more interstitial fibrosis than wild-type mice on the same diet (Figure 7D and E). This suggests that knocking out megalin leads to permanent and progressive chronic kidney disease (CKD) in male mice fed a WD.

**Figure 8.**
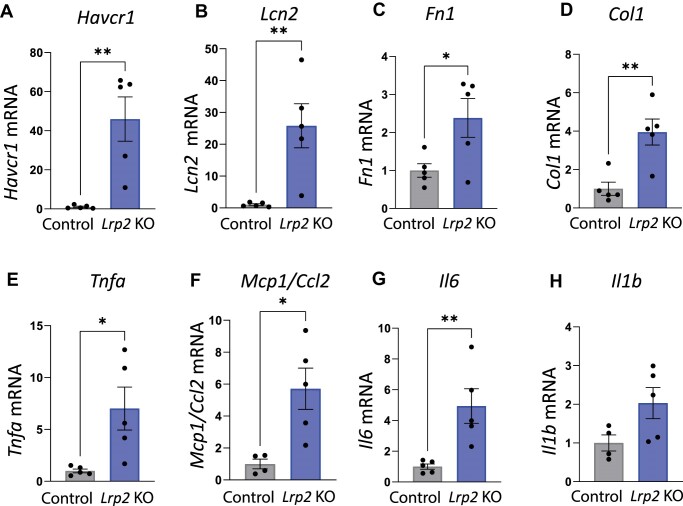
Quantitative real-time reverse transcriptase polymerase chain reaction of control *Lrp2* KO mice following Western diet. Kidneys were collected from control and *Lrp2* KO mice sacrificed after Western diet and processed for qRT-PCR analysis for injury markers (A) *Havcr1*, (B) *Lcn2*, (C) *Fn1*, (D) *Col1*, (E) *Tnfa*, (F) *Mcp1*, (G) *Il6*, and (H) *Il1b. P*-values denoted by asterisks: *≤0.05, **≤0.01. Data analyzed by an unpaired *t*-test.

We used qRT-PCR to assess expression of injury marker transcripts in kidneys harvested from male *Lrp2* KO mice following WD (Figure 8). *Lrp2* KO mice had significantly increased transcript levels of acute kidney injury markers, kidney injury molecule-1 (KIM-1; *Havcr1*), and neutrophil gelatinase-associated lipocalin (NGAL; *Lcn2*), compared with control mice. *Lrp2* KO mice also had significantly increased levels of fibrosis markers fibronectin 1 (*Fn1*) and collagen type 1 (*Col1*), as well as significantly increased levels of inflammation markers tumor necrosis factor alpha (*Tnfa*), monocyte chemoattractant protein-1 (*Mcp1*), and interleukin 1 (*Il6*). Though not quite significant (*P *= 0.074), there was also a trend toward increased transcript levels of inflammation marker interleukin b (*Il1b*) compared to control mice (Figure 8).

### Improved Glucose Tolerance and Protection in WD-fed Female *Lrp2* KO Mice

**Figure 9.**
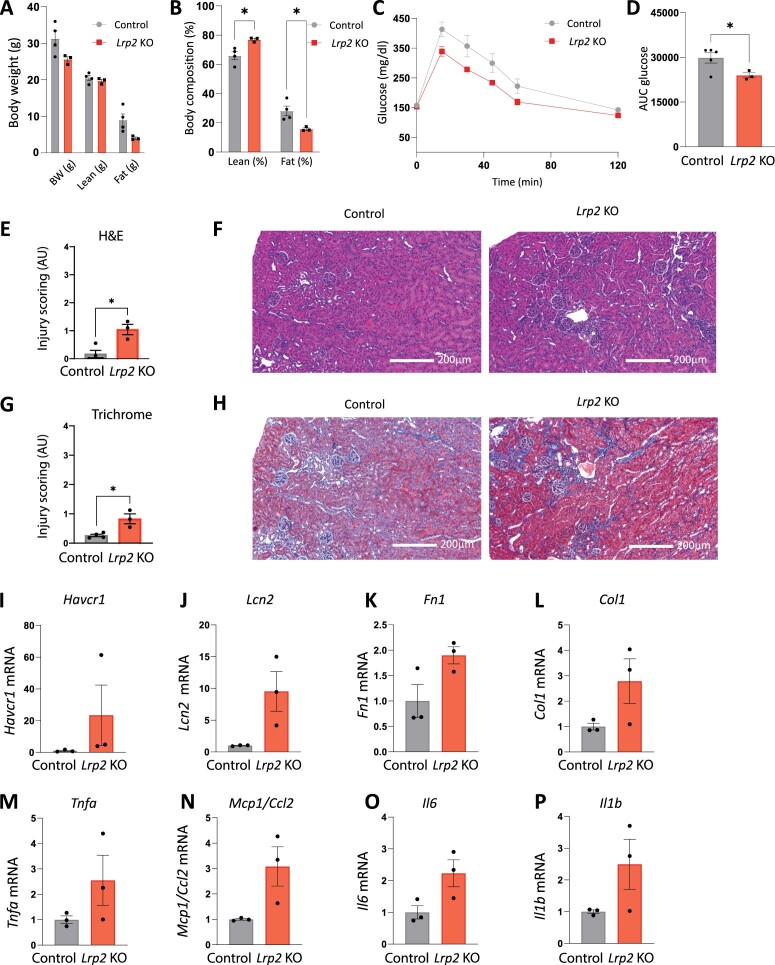
Effects of Western diet on glucose tolerance, metabolic parameters, and kidney injury in female *Lrp2* KO mice. Control and *Lrp2* KO mice were placed on a Western diet for 4 wk and analyzed as in Figure 2. Body weight (A) and composition (B) of control and *Lrp2* KO mice are plotted. Data analyzed by an unpaired *t*-test with multiple comparisons correction (Holm-Sidak). (C) Temporal changes in plasma glucose following intraperitoneal injection of 1.5 mg/kg glucose in fasted control and *Lrp2* KO mice. AUC for glucose calculated in panel C is shown in (D). H&E scoring of stained kidney sections from mice on WD shown in (E) and Masson’s Trichrome scoring of stained kidney sections from mice on WD shown in (G). H&E and Masson’s Trichrome stained sections were scored in a blinded manner as described in the “Methods” section. Representative H&E (F) and Masson’s Trichrome (H) stained kidney sections from control and *Lrp2* KO mice after Western diet. qRT-PCR analysis for injury markers (I) *Havcr1*, (J) *Lcn2*, (K) *Fn1*, (L) *Col1*, (M) *Tnfa*, (N) *Mcp1*, (O) *Il6*, and (P) *Il1b*. Data analyzed by an unpaired *t*-test.

We performed similar analyses to those above on a small cohort of female control and *Lrp2* KO mice. Similar to male *Lrp2* KO mice, female *Lrp2* KO mice exhibit a modest reduction of SGLT2 protein expression (Figure S2). Like male mice, female *Lrp2* KO mice also failed to gain weight on WD, and exhibited a high lean-to-fat mass ratio (Figure 9A, B). Female *Lrp2* KO mice on both RC (Figure S3C and D) and WD (Figure 9C and D) also exhibited improved glucose tolerance compared to control mice. In contrast to male *Lrp2* KO mice on WD, blood chemistry of female mice was largely normal, other than reductions in HCO_3_ and Ca^2+^ (Table 2). The histology of female kidney sections following WD revealed only slightly increased kidney injury in female *Lrp2* KO mice on RC (Figure S4) and on WD compared with control females (Figure 9E-H). qRT-PCR analysis also revealed increased transcript levels of kidney injury biomarkers in female *Lrp2* KO mice on WD compared with control females, although the changes were smaller than those in male *Lrp2* KO mice, and in this small cohort, none reached significance (Figure 9I-P). There were no significant differences in metabolic parameters between control and *Lrp2* KO female mice on either RC or WD, except for activity (Figure S5). RER in *Lrp2* KO female mice trended lower in the light cycle on both RC (Figure S5B) and WD (Figure S5G).

**Table 2.** iSTAT Blood Chemical Analysis of Control and *Lrp2* KO Female Mice on Regular Chow and Western Diet

Average iSTAT Values	*n*	Na^+^ (mmol/L)	K^+^ (mmol/L)	Cl^−^ (mmol/L)	HCO_3_ (mmol/L)	Ca^2+^ (mmol/L)	Hgb (g/dL)	BUN (mmol/L)	Crea (mg/dL)	Hct (%)
Female control (RC)	4	144.3 [0.479]	4.425 [0.085]	114.3 [0.479]	22.75 [0.250]	1.233 [0.033]	12.48 [0.170]	25.75 [1.250]	<0.2	36.75 [0.479]
Female KO (RC)	2	143.5 [2.500]	4.15 [0.050]	113 [2.000]	21 [2.000]	1.18 [0.000]	11.4 [0.500]	30.5 [6.500]	<0.2	33.5 (*P *= 0.096) [1.500]
Female control (WD)	4	144.3 [0.479]	4.450 [0.185]	111.5 [1.041]	25.25 [1.109]	1.333 [0.014]	13.25 [0.371]	19.00 [2.041]	<0.2	39.00 [1.080]
Female KO (WD)	3	142.3 [0.667]	4.300 [0.058]	114.7 [0.882]	19.67* [0.333]	1.210* [0.025]	11.67 [0.694]	19.00 [2.082]	<0.2	34.33 [2.028]

Values are means. Data analyzed by an unpaired *t*-test with multiple comparisons correction (Holm-Sidak). *P*-values denoted by asterisks: *≤0.05. *P*-values <0.1 are noted in parentheses. SEM noted in brackets.

## Discussion

Our studies confirm the complex role for megalin in maintaining kidney and systemic health. We found that both male and female *Lrp2* KO mice had modestly reduced SGLT2 levels that translated to improved glucose tolerance. Additionally, total body weight and fat percentage in *Lrp2* KO mice fed a WD were significantly lower than control. On the other hand, male *Lrp2* KO mice fed a WD developed severe kidney damage accompanied by aberrant blood chemistry. Kidney function in female *Lrp2* KO mice on WD appeared unchanged and kidney damage was considerably milder. While the critical role of PT gluconeogenesis in maintaining glucose levels under starvation conditions is well known, our study demonstrates a striking role for megalin expression in the systemic response to WD.

The metabolic phenotype of *Lrp2* KO mice on RC and WD strongly resembles that of SGLT2 (*Slc5a2*)-deleted mice and SGLT2i-treated mice.^32^,^37^ The glycemic protection we observed in *Lrp2* KO mice is comparable to that observed in *Slc5a2* KO mice,^32^ in canagliflozin-treated wild-type mice,^37^ and in *Sweet Pee* mice that carry a truncation mutation in the *Slc5a2* gene.^41^ Moreover, plasma glucose levels were not reduced in *Lrp2* KO mice, replicating observations in *Sweet P*ee and Slc5a2 KO mice.^32^,^41^  *Lrp2* KO mice also had increased fluid intake as previously observed when Slgt2 expression or function is impaired.^32^,^37^,^41^ Inactivation or deletion of SGLT2 reduces RER and shifts metabolism toward lipid utilization.^32^,^37^ Although RER in *Lrp2* KO mice on RC or WD trended lower than in controls, it did not reach statistical significance. Nevertheless, *Lrp2* KO mice on WD exhibited lower body fat accumulation than controls, similar to observations in diabetic *Slc5a2* KO and *Sweet Pee* mice.^32^,^41^ Lower body fat accumulation has been inferred to mean that animals are in a catabolic state, though we have not confirmed this in our mice.^41^ The less dramatic effect of *Lrp2* KO on RER may reflect the residual activity of SGLT2 in this model.

Surprisingly, despite their lower weight gain and fat accumulation, male *Lrp2* KO mice on WD developed significant kidney injury. Masson’s Trichrome staining revealed significant levels of fibrosis in male *Lrp2* KO mice on WD compared to control mice on the same diet, which had virtually no fibrosis. On the other hand, PT cells in control mice on WD developed more numerous vacuoles compared with *Lrp2* KO mice. This remarkable difference in injury profile between megalin-expressing and megalin-depleted mice may explain why *Lrp2* KO is protective in some disease settings and harmful in others.^25^,^26^,^28^,^29^ In this regard, our data are consistent with a previous study that found reduced vacuolization in uninephrectomized *Lrp2* KO mice fed a high-fat diet compared to similarly treated control mice.^42^ Presumably in the added presence of high levels of refined sugars, loss of megalin expression becomes deleterious to kidney health. We have found differential expression of genes involved in fructose metabolism in *Lrp2* KO OK cells compared to control OK cells, though these differences have not been confirmed in our mouse model.^21^ Differences in fructose metabolism may contribute to the deleterious effects of megalin KO on kidney health following WD.

Interestingly, while glycemic protection in female *Lrp2* KO mice was similar to that in males, female mice did not exhibit extensive kidney injury following WD. Indeed, women and female mice are generally protected against the onset of diabetes, and female mice are resistant to WD-induced weight gain. Increased oxidative stress and mitochondrial dysfunction contribute to renal injury in mice on high-fat diets.^43^ Women have also been shown to have higher mitochondrial spare respiratory capacity and lower reactive oxygen species (ROS) production than men in most tissues.^44^^-^ ^46^Additionally, PT energy expenditure in female rodents is thought to be lower than in males due to a greater reliance on the distal convoluted tubule for sodium reabsorption.^47^,^48^ Together, these factors may contribute to the enhanced protection of female *Lrp2* KO mice against WD-induced kidney injury. Understanding the relationship between diet and *Lrp2*-mediated sex-dependent protection or injury may provide novel targets for managing obesity and T2D.

Our study has some limitations that limit the extent of conclusions we can draw. The cohort of female mice that we tested was small, and we may have missed differences that would be evident in a larger group. Additionally, while our studies demonstrate a substantial role for megalin expression in maintaining PT function, our work here does not address how *Lrp2* KO alters SGLT2 expression or activity, or how megalin defends against WD-induced kidney disease. We demonstrate an increase in proinflammatory cytokines in *Lrp2* KO mice, and inflammation is strongly implicated in the development of tubular injury and fibrosis (PMC6792167). How megalin affects inflammation, and whether this inflammation is causing the kidney injury, or a result of underlying tubular injury, will require investigation in future studies.

Despite these limitations, our studies raise new fundamental questions that remain to be resolved. Why is the apparently modest reduction in SGLT2 levels in *Lrp2* KO mice sufficient to reproduce the effect of SGLT2 deletion? Although we did not observe any reduction in SGLT1 expression based on quantitation of *Slc5a1* transcript levels, it is possible that SGLT1 protein levels are also reduced in *Lrp2* KO mice and contribute to the phenotype. A second question is how megalin mediates the complex response to WD. While the primary function ascribed to megalin is the endocytic retrieval of filtered proteins, KO of megalin in PT cells also affects transcription, ion transport, and cell metabolism.^21^ Understanding the interplay between these diverse functions may explain the balance of protective and deleterious effects of *Lrp2* KO in response to dietary conditions.

## Supplementary Material

zqae026_Supplemental_File

## Data Availability

The data underlying this article are available in Figshare and can be accessed at doi: 10.6084/m9.figshare.25843504.
