# Supplementary material for: Megalin Knockout Reduces SGLT2 Expression and Sensitizes to Western Diet-induced Kidney Injury
Source: Function (Oxf). 2024 May 20;5(4):zqae026. doi: 10.1093/function/zqae026 (PMC11237895; doi:10.1093/function/zqae026)
Supplement: zqae026_Supplemental_File [file zqae026_supplemental_file.pdf]

Table S1. qPCR Primers

Gene	Forward	Reverse
mCol1a1	*5'-ATCTCCTGGTGCTGATGGAC-3'*	*5'-ACCTTGTTTGCCAGGTTCAC-3'*
mFn1	*5'-CGAGGTGACAGAGACCACAA-3'*	*5'-CTGGAGTCAAGCCAGACACA-3'*
mHavcr1	*5'-TTCAGGAAGCTGAGCAAACAT-3'*	*5'-CCCCAACATGTCGTTGTGATT-3'*
mIl1b	*5'-TGCCACCTTTTGACAGTGATG-3'*	*5'-ATGTGCTGCTGCGAGATTTG-3'*
mIl6	*5'-CTTGGGACTGATGCTGGTG-3'*	*5'-TCCACGATTTCCCAGAGAAC-3'*
mLcn2	*5'-CCATCTATGAGCTACAAGAGAACAAT-3'*	*5'-TCTGATCCAGTAGCGACAGC-3'*
mMcp1	*5'-CACTCACCTGCTGCTACTCA-3'*	*5'-GCTTGGTGACAAAAACTACAGC-3'*
mPpia	*5'-AGGTGAAAGAAGGCATGAAC-3'*	*5'-ACAGTCGGAAATGGTGATCT-3'*
mTnfa	*5'-TCGTAGCAAACCACCAAGTG-3'*	*5'-CCTTGAAGAGAACCTGGGAG-3'*

Supplementary Figure Legends

Figure S1. Injury scoring of H&E and Masson's trichrome stained kidney sections from male mice on regular chow. H&E and Masson's Trichrome stained sections were scored in a blinded manner as described in Methods. (A) H&E scoring and (C) Masson's Trichrome scoring of stained kidney sections from mice on regular chow. Data analyzed by unpaired t-test. Representative H&E and Masson's Trichrome stained sections are shown in (B) and (D), respectively.

Figure S2. Megalin and SGLT2 expression in female control and *Lrp2* KO mice. Western blot (A) of mouse kidney lysates from control and *Lrp2* KO female mice on regular chow probed to detect megalin and SGLT2. (B) Quantitation of SGLT2 expression in panel A. Data analyzed by unpaired t-test. (C) Cortical kidney sections of control and *Lrp2* KO mice were stained to reveal SGLT2 (green). Merged panels below show megalin (red) and nuclei (blue) in the panels below each image to verify efficient KO. SGLT2 exposures and processing were identical for all images. Megalin staining intensity in the *Lrp2* KO image was selectively enhanced to enable better comparison of expressing vs non-expressing cells. Scale bars: 25 µm.

Figure S3. Body composition and glucose tolerance test of female *Lrp2* KO mice on regular chow. Body weight (A) and composition (B) control and *Lrp2* KO mice are plotted. Data analyzed by unpaired t-test with multiple comparisons correction (Holm-Sidak). (C) Temporal changes in plasma glucose following intraperitoneal injection of 2.0 mg/kg glucose in fasted control and *Lrp2* KO mice. Area under the curve (AUC) for glucose calculated in panel C is shown in (D). (P-values denoted by asterisk: * = ≤0.05.) Data analyzed by unpaired t-test.

Figure S4. Injury scoring of H&E and Masson's trichrome staining of kidney sections from female mice on regular chow. (A) H&E and (C) Masson's Trichrome stained kidney sections from mice on regular chow were scored in a blinded manner as described in Methods. (P-values denoted by asterisks: * = ≤0.05, ** =≤0.01.) Data analyzed by unpaired t-test. Representative H&E and Masson's Trichrome stained sections are shown in (B) and (D), respectively.

Figure S5. Metabolic cage data of female control and *Lrp2* KO mice on regular chow and Western diet. Control and *Lrp2* KO female mice fed a regular chow or Western diet were evaluated in metabolic cages for 48h. Traces for each parameter are shown in the left graph of each panel (gray bars denote dark cycles), and average data for each cycle and total over the 48h period are plotted on the right. Regular chow (A) Activity; (B) Respiratory Exchange Ratio; (C) Energy expenditure; (D) Drinking; (E) Feeding. Western diet (F) Activity; (G) Respiratory Exchange Ratio; (H) Energy expenditure; (I) Drinking; (J) Feeding.(P-values denoted by asterisk: * =≤0.05, ** = ≤0.01.) Data analyzed by unpaired t-test with multiple comparisons correction (Holm-Sidak).

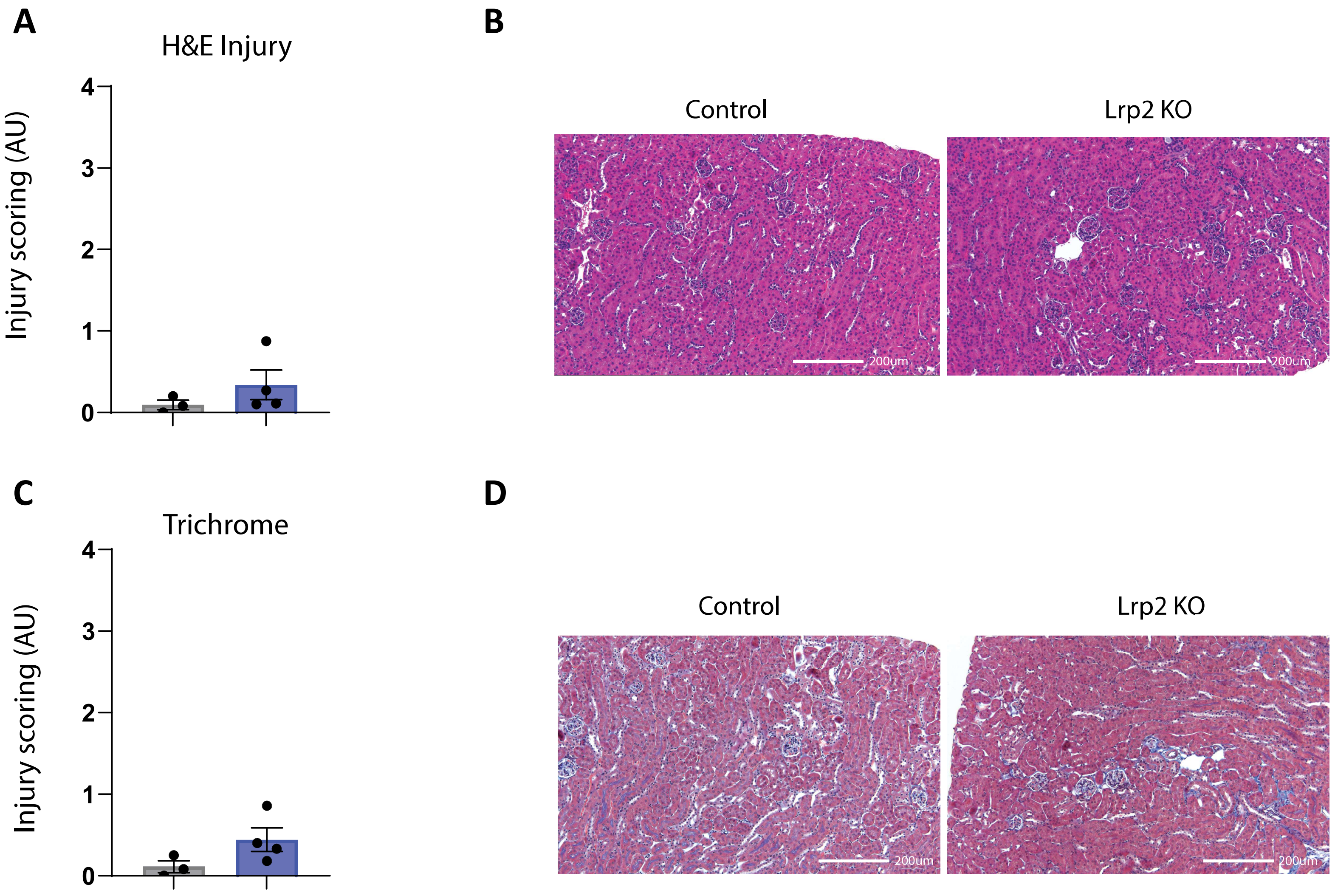
A
H&E Injury
Injury scoring (AU)
0
1
2
3
4
B
Control
Lrp2 KO
200um
200um
C
Trichrome
Injury scoring (AU)
0
1
2
3
4
D
Control
Lrp2 KO
200um
200um

Supp Fig 1

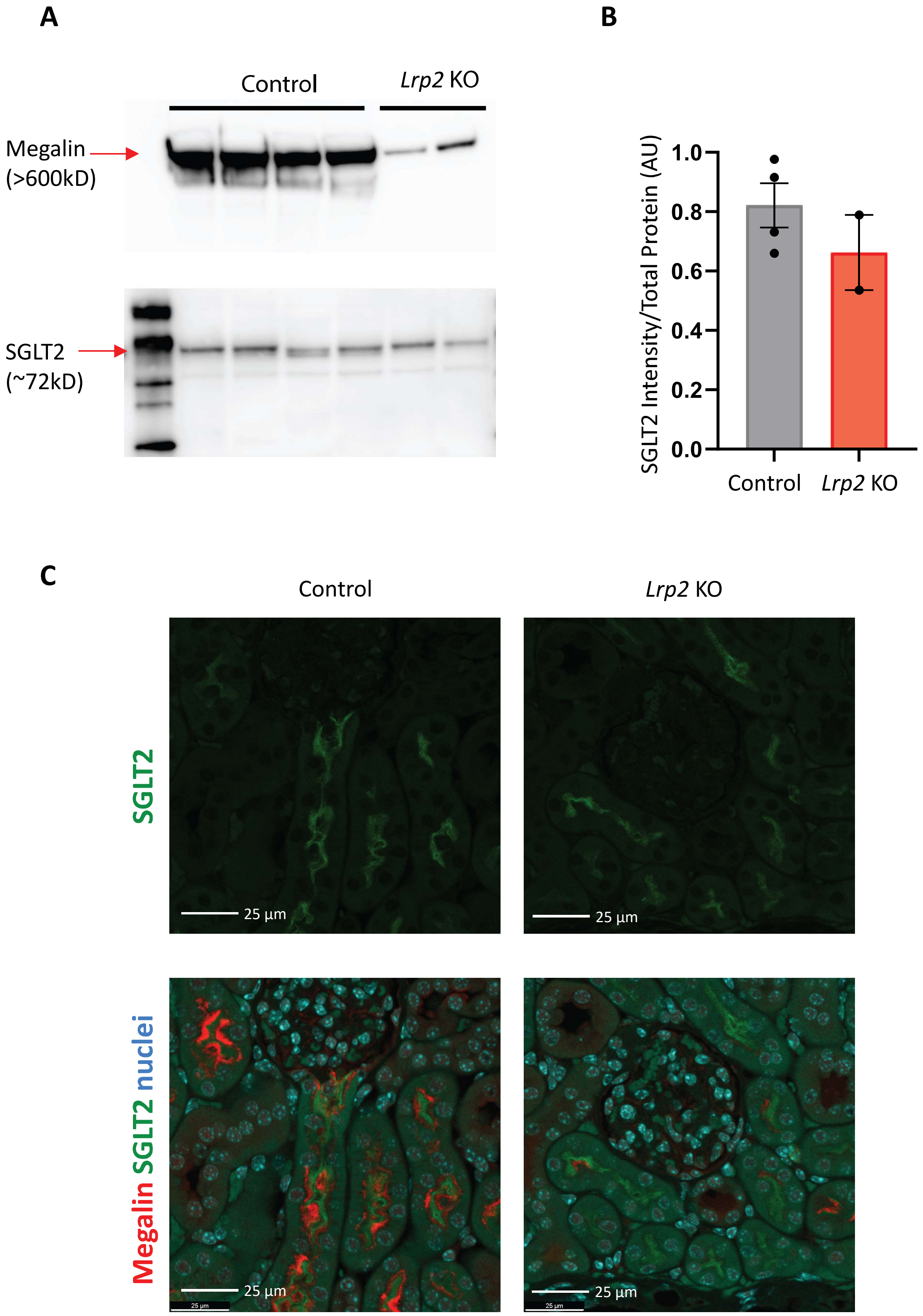
A
Control
Lrp2 KO
Megalin
(>600kD)
SGLT2
(~72kD)
B
SGLT2 Intensity/Total Protein (AU)
1.0
0.8
0.6
0.4
0.2
0.0
Control
Lrp2 KO
C
Control
Lrp2 KO
SGLT2
Megalin SGLT2 nuclei
25 µm

Supp Fig 2

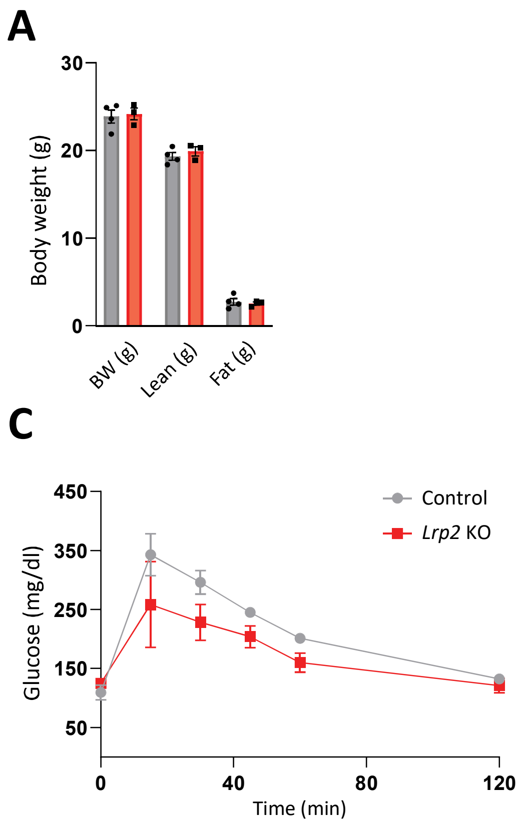

A
30
20
10
0
Body weight (g)
BW (g)
Lean (g)
Fat (g)
B
100
80
60
40
20
0
Body composition (%)
Lean (%)
Fat (%)
C
450
350
250
150
50
Glucose (mg/dl)
0
40
80
120
Time (min)
Control
Lrp2 KO
D
45000
30000
15000
0
AUC glucose
*
Control
Lrp2 KO

Supp Fig 3

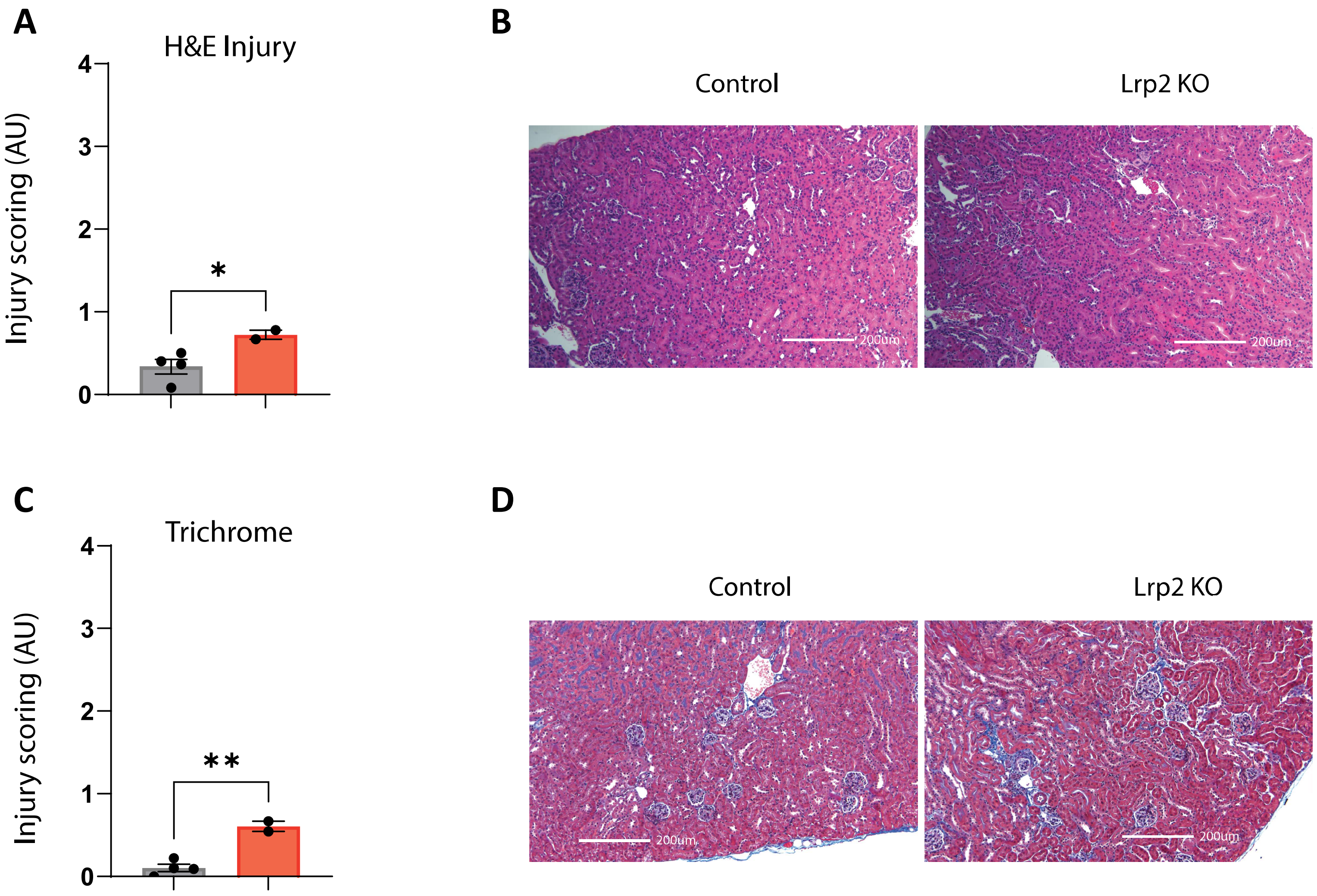
A
H&E Injury
Injury scoring (AU)
4
3
2
1
0
*
B
Control
Lrp2 KO
200um
200um
C
Trichrome
Injury scoring (AU)
4
3
2
1
0
**
D
Control
Lrp2 KO
200um
200um

Supp Fig 4

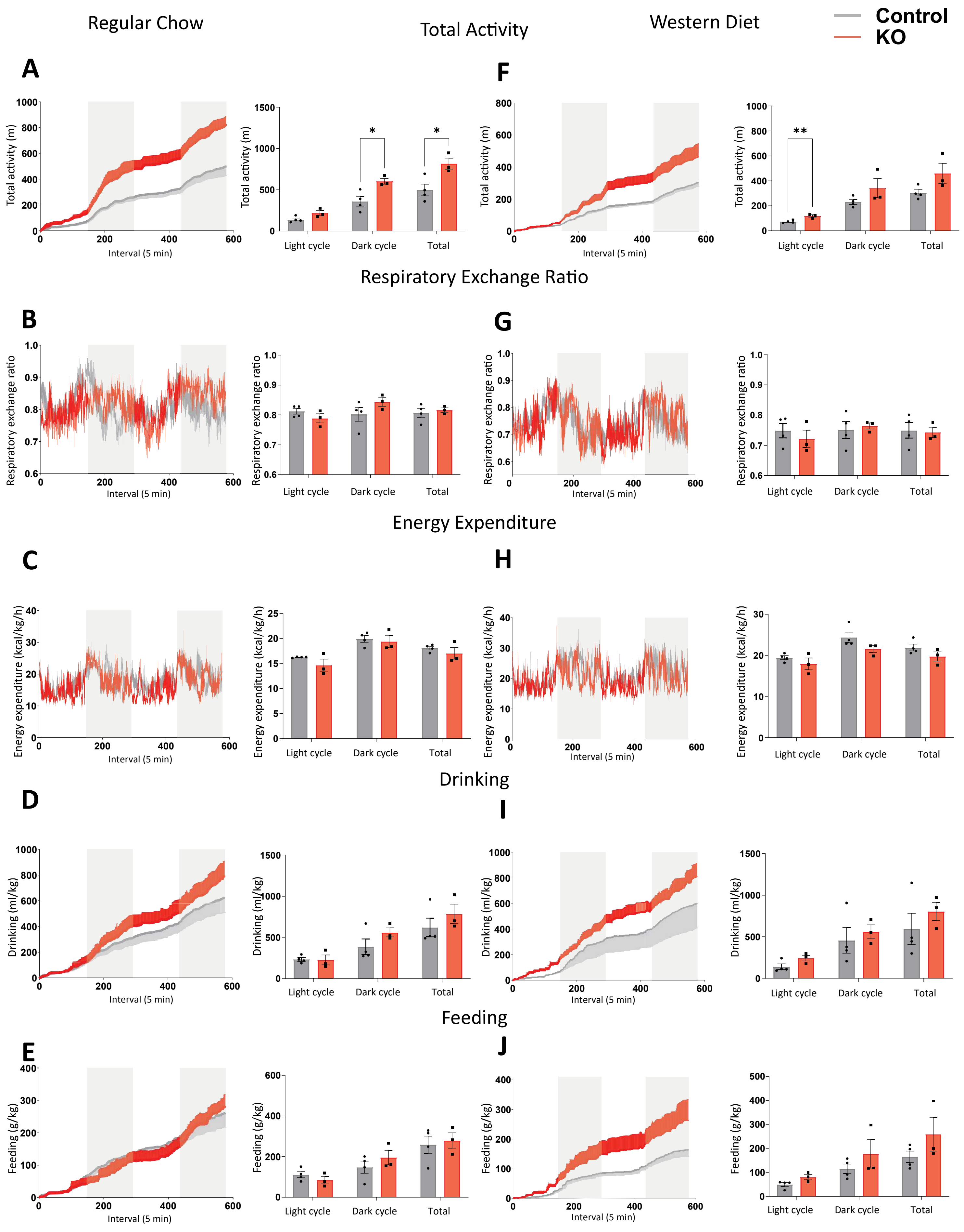

Regular Chow
Total Activity
Western Diet
Control
KO
A
F
Total activity (m)
Interval (5 min)
Light cycle
Dark cycle
Total
Respiratory Exchange Ratio
B
G
Respiratory exchange ratio
Energy Expenditure
C
H
Energy expenditure (kcal/kg/h)
Drinking
D
I
Drinking (ml/kg)
Feeding
E
J
Feeding (g/kg)

Supp Fig. 5
